# Granulin-Epithelin Precursor Is an Oncofetal Protein Defining Hepatic Cancer Stem Cells

**DOI:** 10.1371/journal.pone.0028246

**Published:** 2011-12-16

**Authors:** Phyllis Fung Yi Cheung, Christine Kei Chin Cheng, Nicholas Chun Lim Wong, Jenny Chung Yee Ho, Chi Wai Yip, Vincent Chi Hang Lui, Annie Nga Yin Cheung, Sheung Tat Fan, Siu Tim Cheung

**Affiliations:** 1 Department of Surgery, The University of Hong Kong, Hong Kong, China; 2 Center for Cancer Research, The University of Hong Kong, Hong Kong, China; 3 Department of Pathology, The University of Hong Kong, Hong Kong, China; 4 State Key Laboratory for Liver Research, The University of Hong Kong, Hong Kong, China; Yonsei University College of Medicine, Republic of Korea

## Abstract

**Background and Aims:**

Increasing evidence has suggested that hepatocellular carcinoma (HCC) might originate from a distinct subpopulation called cancer stem cells (CSCs), which are responsible for the limited efficacy of conventional therapies. We have previously demonstrated that granulin-epithelin precursor (GEP), a pluripotent growth factor, is upregulated in HCC but not in the adjacent non-tumor, and that GEP is a potential therapeutic target for HCC. Here, we characterized its expression pattern and stem cell properties in fetal and cancerous livers.

**Methods:**

Protein expression of GEP in fetal and adult livers was examined in human and mouse models by immunohistochemical staining and flow cytometry. Liver cancer cell lines, isolated based on their GEP and/or ATP-dependent binding cassette (ABC) drug transporter ABCB5 expression, were evaluated for hepatic CSC properties in terms of colony formation, chemoresistance and tumorigenicity.

**Results:**

We demonstrated that GEP was a hepatic oncofetal protein that expressed in the fetal livers, but not in the normal adult livers. Importantly, GEP+ fetal liver cells co-expressed the embryonic stem (ES) cell-related signaling molecules including β-catenin, Oct4, Nanog, Sox2 and DLK1, and also hepatic CSC-markers CD133, EpCAM and ABCB5. Phenotypic characterization in HCC clinical specimens and cell lines revealed that GEP+ cancer cells co-expressed these stem cell markers similarly as the GEP+ fetal liver cells. Furthermore, GEP was shown to regulate the expression of ES cell-related signaling molecules β-catenin, Oct4, Nanog, and Sox2. Isolated GEP^high^ cancer cells showed enhanced colony formation ability and chemoresistance when compared with the GEP_low_ counterparts. Co-expression of GEP and ABCB5 better defined the CSC populations with enhanced tumorigenic ability in immunocompromised mice.

**Conclusions:**

Our findings demonstrate that GEP is a hepatic oncofetal protein regulating ES cell-related signaling molecules. Co-expression of GEP and ABCB5 further enriches a subpopulation with enhanced CSC properties. The current data provide new insight into the therapeutic strategy.

## Introduction

Hepatocellular carcinoma (HCC), the third leading cause of cancer mortality worldwide, is a highly malignant disease with no effective treatment currently [1]. While the molecular mechanisms of HCC pathogenesis remain largely unknown, HCC is considered a heterogeneous disease due to its multiple molecular profiles and varied clinical outcomes [2]. The heterogeneous nature of HCC and the lack of appropriate biomarkers have impeded patient prognosis and treatment.

For years, tumor cell heterogeneity has been explained by the clonal evolution model [3]. However, recent evidence suggests that tumors are hierarchically organized with a distinct subpopulation called cancer stem cells (CSCs) lying at the apex of the hierarchy. These cells are not only responsible for tumor initiation and progression, but also endowed with stem cell properties, including self-renewal, differentiation capacity and chemoresistance [4]. Although existing therapies can initially eliminate the bulk population of tumor, the stem cell properties of CSCs enable them to survive and repopulate the tumor, resulting in disease relapse [5].

The concept of CSCs has been documented in diverse malignancies including breast [6], colon [7] and liver cancers [8], [9]. Despite recent advances in hepatic CSC identification, precise origin of these cells remains largely unknown. Tumorigenesis and embryogenesis are known to share many common properties including cellular plasticity, dynamic cell motility [10] and convergence of signaling pathways, suggesting a possible link between embryonic and cancer cells [11]. Interestingly, the concept of CSC is notably similar to the “embryonal rest” theory, which was proposed based on the histological similarities of embryonic and cancerous tissues, suggesting that adult tissues contain embryonic remnants that normally lie dormant, but can be activated to become cancerous [12]. Therefore, identification of key molecule that underlie the commonality of embryonic stem (ES) cells and tumor cells might result in new therapeutic strategies to suppress the malignant transformation of normal stem cells, or eradicate the CSCs.

Granulin-epithelin precursor (GEP, also named progranulin, proepithelin, acrogranin, or PC-derived growth factor) is a pluripotent growth factor regulating fetal development [13], tissue repair [14] and tumorigenesis in various cancers [15]. It was found to regulate developmental events including cavitation in preimplantation mouse embryos [16] and male-specific brain differentiation of the hypothalamus of neonatal rats [17]. Previously, we have demonstrated GEP overexpression in the majority of human HCC specimens [18], [19] and its role in regulating HCC cell proliferation, invasion and tumorigenicity [19]. Moreover, neutralization of GEP could inhibit the growth of established HCC [20]. Despite considerable interest in its dual roles in embryogenesis and tumorigenesis, information for its expression pattern and biological functions in livers is still scarce.

In this study, we report for the first time that GEP is a hepatic oncofetal protein that regulates the expression of stem cell-related signalling molecules β-catenin, Oct4, Nanog and Sox2. Functional studies confirmed that GEP-expressing cells possessed greater ability in colony formation and chemoresistance. Furthermore, cancer cells co-expressing GEP and ATP-dependent binding cassette (ABC) B5 demonstrated enhanced tumorigenic ability in immunocompromised mice. Our findings are crucial not only for the understanding of fetal liver development, but also for constructing specific therapies targeting GEP and ABCB5 to eradicate the chemoresistant CSCs in HCC patients.

## Materials and Methods

### Cell assays

Human liver cancer cell lines, Hep3B and HepG2, were purchased from American Type Culture Collection (Manassas, VA) and cultured as previously described [19], [20]; while Huh7 was purchased from Health Science Research Resources Bank (Osaka, Japan) and maintained in Dulbecco's modified Eagle medium (DMEM) supplemented with 10% heat-inactivated fetal bovine serum (Invitrogen, Carlsbad, CA). Stable transfectants for GEP overexpression and suppression have been described [19]. GEP blockage in Hep3B was performed by incubating the cells with or without 50 µg/ml anti-GEP monoclonal antibody A23 (homemade, previously described [20] or mouse IgG isotype control antibody (Sigma-Aldrich, St. Louis, MO) for 24 h.

### Clinical specimens

The study protocol was approved by the Institutional Review Board of the University of Hong Kong/Hospital Authority Hong Kong West Cluster (HKU/HA HKW IRB). Six patients underwent curative partial hepatectomy or liver transplantation for HCC at Queen Mary Hospital, Hong Kong, were recruited with written informed consent to the study. The patients had been diagnosed with primary HCC and confirmed by pathological examinations. The age of the patients ranged from 42 to 67 years, with a median age of 60 years. There were 4 men and 2 women, and all were hepatitis B virus carriers. The size of the tumors ranged from 3.5 to 19.5 cm, with a median size of 8.3 cm. Notably, each specimen has limited number of cells (small research specimen to ensure no interference to pathological examination) and therefore not sufficient for the complete panel of stem cell marker expression analysis. The expression data of each marker presented the average data from at least four samples. The normal liver specimen was obtained from organ donor during transplantation operation, and the donor had no underlying liver disease and was negative in the hepatitis B serology test. The fetal liver specimen was obtained from retrieval of archived formalin fixed paraffin embedded tissue block taken during routine pathological examination of the abortus after spontaneous miscarriage at 10 weeks 6 days. Ethics approval for the use of archived fetal tissues identified from a computer database has been obtained from the Institutional Review Board of the University of Hong Kong/Hospital Authority Hong Kong West Cluster (HKU/HA HKW IRB).

### Mouse specimens

Livers from adult, neonatal and fetal ICR mice were collected and the study protocol was approved by the Committee on the Use of Live Animals in Teaching and Research at the University of Hong Kong (Approval Reference No. CULATR 1968-09). For isolation of mouse hepatocytes, livers were minced and digested with type IV collagenase (Sigma-Aldrich, St. Louis, MO). After filtering and lysis of red blood cells, cells were counted for immunofluorescence staining and flow cytometric analysis (FACSCalibur, BD Biosciences, San Jose, CA). Cells isolated from embryonic livers were stained with anti-AFP antibody (R&D systems, Minneapolis, MN) and anti-albumin antibody (R&D systems) to distinguish hepatocytes for subsequent characterization of GEP-expressing cells.

### Immunohistochemistry

Immunohistochemistry was performed on formalin-fixed paraffin-embedded sections with Dako Envision Plus System (Dako, Carpinteria, CA) following the manufacturer's instruction with modifications as described [18], [19].

### Immunofluorescence staining and flow cytometric analysis

For expression of GEP, ABCB5, β-catenin, Oct4, Sox2,Nanog and DLK1, cells were permeabilized with ice-cold 0.1% saponin and then incubated with FITC-conjugated mouse anti-human GEP (described previously [20]), unconjugated goat anti-human ABCB5 (Everest Biotech Ltd, Oxfordshire, UK), Alexa Fluor 647-conjugated mouse anti-human β-catenin, PerCP-Cy5.5-conjugated mouse anti-human Oct4, Alexa Fluor 647-conjugated mouse anti-Sox2, PE-conjugated mouse anti-human Nanog (BD Biosciences), unconjugated rat anti-DLK-1 (MBL International, Woburn, MA), or equal amount of corresponding isotype control antibodies. For ABCB5 and DLK1, cells were incubated with NorthernLights (NL) 557-conjugated donkey anti-goat IgG secondary antibody or NL637-conjugated goat anti-Rat IgG secondary antibody, respectively, prior to staining with anti-GEP antibody. For CD133 and EpCAM surface expression, cells were stained with APC-conjugated mouse anti-human CD133 (Miltenyi Biotec, Bergisch Gladbach, Germany), APC-conjugated mouse anti-human EpCAM (BD Biosciences) or equal amount of corresponding isotype control antibodies, prior to permeabilization and staining with anti-GEP antibody.

### Cell sorting

Isolation of GEP- and/or ABCB5-expressing cells was performed by magnetic activated cell sorting (Miltenyi Biotec) according to manufacturer's instructions. Briefly, cells were labeled with FITC-conjugated mouse anti-human GEP (previously described [20]) or biotin-conjugated rabbit anti-human ABCB5 (Rockland, Gilbertsville, PA) antibody, and then incubated with anti-FITC or anti-biotin magnetic microbeads (Miltenyi Biotec), followed by magnetic separation. Note that cells were sorted based on the surface expression of GEP and ABCB5, but not on their intracellular expression, because permeabilization was not feasible to keep the cells viable for subsequent functional assays. After cell isolation, 2 million of each sorted population were collected to assess the cell viability by trypan blue staining and purity by flow cytometry using mouse anti-human GEP (R&D systems) or goat anti-human ABCB5 (Everest Biotech) antibody recognizing epitopes different from those antibodies used for cell sorting. Cells were then stained with PE-conjugated rabbit anti-mouse or NL557-conjugated donkey anti-goat IgG secondary antibody for GEP or ABCB5, respectively. For the evaluation of cell purity by flow cytometry, sorted cells were permeabilised prior to staining so that the total GEP and ABCB5 expression of the sorted populations could be determined. Post-sorting analysis typically indicated purities of >80% with minimal cell death (<10%).

### Doxorubicin accumulation and apoptosis

Cells were incubated with or without 0.5 µg/ml doxorubicin for 24 hr. Intracellular doxorubicin accumulation was analyzed by flow cytometry at FL2 spectrum; while cell apoptosis was determined by Annexin V-FITC (FL1) and propidium iodide (PI) (FL2) staining and flow cytometric analysis.

### Colony formation assay

Freshly isolated cells were seeded at a density of 1000 cells/well and allowed to grow for 10 days. Colony formation was assessed by a colorimetric assay using crystal violet (Sigma Aldrich).

### *In vivo* tumorigenicity experiments

BALB/c athymic nude mice of 5 weeks old were used to test the *in vivo* tumorigenic potential of the cells. The study protocol was approved by the Committee on the Use of Live Animals in Teaching and Research at the University of Hong Kong. Various numbers of cells were inoculated subcutaneously at the dorsal region of the trunk of each animal. Mice were sacrificed between 8 and 16 weeks post-injection, at which tumors were harvested for further examination. Those mice injected with HCC cells but with no sign of tumor burden were generally terminated 5 months after cell injection.

### Statistical Analyzes

All data were expressed as mean values ± standard deviation (SD) from at least three independent experiments. Differences between groups were assessed by the Student's t test. A probability (p)<0.05 was considered significantly different. All analyzes were performed using the statistical software GraphPad Prism for Windows, Version 3.00 (GraphPad Software, CA, USA).

## Results

### GEP is a hepatic oncofetal protein

**Figure 1:**
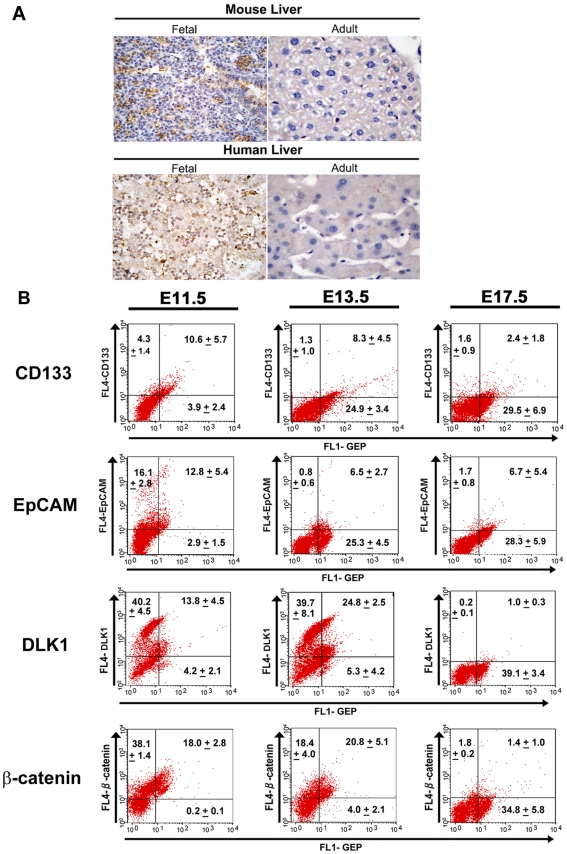
GEP expression in mouse and human livers. (A) Immunohistochemical analysis showing GEP protein in fetal livers of mouse (embryonic day 17.5) and human (10 weeks 6 days), but not in adult livers (Magnification ×400). (B) Flow cytometric analyzes demonstrating the expression of GEP and hepatic stem cell markers in mouse embryonic hepatocytes. The co-expression of GEP and hepatic stem cell markers CD133, EpCAM, DLK1 and β-catenin were performed by quadruple-color flow cytometry, gating on the AFP+ and/or albumin+ hepatocytes of embryonic livers. Cells co-expressing the respective markers were shown in the upper right quadrant of dot plots. Data are expressed as mean percentage of cells+SD.

Previously, GEP mRNA was reported in the embryonic mouse liver [13], but whether GEP translates into protein and its time of switch-on/-off during liver development remains unknown. Since fetal liver has plenty of hematopoietic stem cells in addition to fetal hepatocytes, we need to identify the cell type(s) expressing GEP. Here, we described for the first time the protein expression pattern of GEP in human and mouse livers. By immunohistochemical staining, GEP protein was detected in mouse and human fetal livers, while adult livers were devoid of GEP-expressing hepatocytes (Figure 1A).

In mouse model, in order to distinguish GEP expression specifically in hepatocytes, analysis of GEP level was performed by triple-color flow cytometry, gating on AFP+ and/or albumin+ for hepatocytes at all developmental stage. Inclusion of AFP+ and/or albumin+ cells in all developmental stages would better reflect the hepatocyte population in the liver because the proportion of AFP+ cells and albumin+ cells were different in embryonic, neonatal and adult livers (Figure S1). GEP expression increased gradually from embryonic day E11.5 (14.8%+6.9%) to E17.5 (30.8+9.2%). Immediately after birth, GEP level decreased abruptly from day 1 (4.4+1.5%) to day7 (1.2+0.4%), and became almost undetectable in adult mouse livers (0.9+0.4%) (Figure S1). Together with our previous findings that GEP expressed in the majority of HCC specimens, but not in the adjacent non-tumor liver tissues [18], [19], we confirmed that GEP was a hepatic oncofetal protein.

To characterize the stem cell signature of GEP+ fetal hepatocytes, we examined the co-expression of GEP and several hepatic stem cell or stem cell-related markers including CD133, EpCAM, DLK1 and β-catenin [21], [22], [23], [24] in the embryonic livers (Figure 1B). Hepatoblasts would have serial changes along developmental stages and give rise to hepatocytes and cholangiocytes. At embryonic day 11.5 (E11.5), expressions of CD133, EpCAM, DLK1 and β-catenin were high in the hepatoblasts and the majority of GEP-positive cells co-expressed all these hepatic stem cell markers. At E13.5, expressions of CD133 and EpCAM started to decline and most of the CD133+ or EpCAM+ cells were observed to co-express GEP. For DLK1 and β-catenin, their expression levels remained high at E13.5 and the majority of GEP-expressing cells were also positive for both markers. At E17.5, the expressions of CD133, EpCAM, DLK1 and β-catenin decreased to low levels, while GEP expression reached the peak at this stage. The discrepancy of expression trends between GEP and these stem cell markers could be attributed to the growth factor properties of GEP. While further investigation is needed to elucidate the mechanism underlying the expression pattern of GEP, the co-expression of GEP with these hepatic stem cell or stem cell-related markers suggests a stem cell phenotype of GEP-expressing cells.

The GEP expression in the mouse hepatocytes was investigated by our home-made anti-GEP antibody A23. The specificity of A23 was examined by western blot (Figure S2 and the result showed that it could recognize the mouse GEP as single band from the mouse embryonic liver protein extract. Moreover, the GEP expression pattern as shown in the western blot was consistent with flow cytometry data in that GEP protein level increased from E11.5 and E13.5 to E17.5, providing consistent data to reflect the GEP expression in the embryonic livers.

### Phenotypic characterization of GEP-expressing cells in HCC

To better characterize if the GEP-expressing cells had the stem cell properties in HCC, we examined the co-expression of GEP and a panel of stem cell markers using flow cytometry in human HCC cell lines Hep3B and Huh7. Markers examined include hematopoietic precursor cell surface markers CD31, CD34, CD117, hepatic precursor cell surface marker CD123, stem cell surface markers CD24, CD29, hepatic CSC surface markers CD44, CD90, CD133, EpCAM, ES cell-related signaling molecules β-catenin, Oct4, Nanog and Sox2, ABC drug transporters ABCC1 and ABCB5. Note that ABC drug transporters were also included because both normal and cancer stem cells expressed higher level of ABC drug transporters, contributing to greater resistance to chemotherapeutic agents than differentiated cells [4], [21]. Among the markers being investigated, GEP+ cells were shown to preferentially co-express with β-catenin, Oct4, Nanog, Sox2, CD133 and EpCAM when compared with GEP- cells in both HCC cell lines Hep3B (Figure S3) and Huh7 (Figure S4). Our findings therefore suggested a stem cell feature of GEP-expressing cells in the both embryonic and cancerous liver cells.

### GEP regulates expression of stem cell markers

We modulated the GEP expression levels in Hep3B cells by transfection approach and investigated if the protein levels of the stem cell markers would be altered. Results showed that up-regulation of GEP level enhanced, while suppression of GEP level decreased the expression of β-catenin, Oct4, Nanog, Sox2 and ABCB5 (Table 1). Modulation on GEP levels did not changed the expression of hepatic stem cell surface markers CD133 and EpCAM. The reason might be due to the fact that the majority of Hep3B cells were positive for CD133 and EpCAM (greater than 95%), thus change of one molecule (e.g. GEP) might not be able to shift the population. While GEP has been previously reported by our group to regulate ABCB5 to mediate chemoresistance in HCC cells [25], the positive correlation between GEP and β-catenin, Oct4, Sox2 and Nanog suggested that GEP might regulate the expression of these ES cell-related signaling molecules to maintain the pluripotency of stem cells.

**Table 1:** GEP regulates stem cell marker expression in liver cancer cell line Hep3B.

	Hep3B parental	GEP Overexpression (FL)	GEP Suppression (sh)
GEP+ (%)	66.8±2.2	85.7±1.8 **	20.1±2.9 ***
β-catenin+ (%)	18.3±1.2	40.5±1.5 ***	11.5±3.1 *
GEP+β-catenin+ (%)	16.8±1.9	37.5±1.3 ***	10.8±1.5 **
Oct4+ (%)	7.9±0.8	11.1±1.3 **	2.4±0.3 **
GEP+Oct4+ (%)	7.4±0.7	10.3±0.9 ***	2.3±0.4 **
Nanog+ (%)	3.8±0.6	3.9±0.1	0.0±0.0 **
GEP+Nanog+ (%)	3.8±0.2	3.8±0.2	0.0±0.0 ***
Sox2+ (%)	1.3±0.3	5.0±0.2 ***	0.4±0.1 *
GEP+Sox2+ (%)	1.1±0.1	4.5±0.1 ***	0.2±0.2 **
CD133+ (%)	94.9±0.9	96.2±1.8	94.8±3.0
GEP+CD133+ (%)	66.1±2.3	75.4±0.2 *	19.4±2.9 ***
EpCAM+ (%)	95.6±0.6	96.4±1.7	95.2±0.4
GEP+EpCAM+ (%)	63.6±1.7	75.3±2.3 ***	19.9±2.3 ***
ABCB5+ (%)	27.8±3.8	72.9±4.5 ***	4.7±1.9 **
GEP+ABCB5+ (%)	27.3±3.4	63.7±5.3 ***	4.4±1.7 **

GEP overexpression increased, while GEP suppression decreased the expression of β-catenin, Oct4, Nanog, Sox2 and ABCB5, but not CD133 and EpCAM.

Cells were dually stained for GEP and stem cell markers. Protein expression of GEP, β-catenin, Oct4, Nanog, Sox2 and ABCB5 was detected by intracellular staining, while that of CD133 and EpCAM was assessed by surface staining, and was then analyzed by flow cytometry. Data are expressed as mean percentage of positive cells ± SD from three independent experiments.

*P<0.05,

**P<0.01,

***P<0.001 when compared with Hep3B control.

To further validate the regulatory role of GEP on the expression of these signaling molecules, GEP blockage by anti-GEP monoclonal antibody A23 was performed in Hep3B cells (Table 2). A23 was found to significantly reduce endogenous GEP levels in Hep3B cells. GEP blockage also significantly suppressed the expression of β-catenin, Oct4, Nanog and Sox2, thereby confirming the regulatory role of GEP on the expression of these stem cell-related signalling molecules.

**Table 2:** GEP blockage by anti-GEP antibodies suppresses the expression of ES cell-related signaling molecules and ABCB5.

	CTL	mIgG	A23
GEP+ (%)	70.3±3.6	67.3±1.1	37.7±6.7 *
β-catenin+ (%)	15.4±2.6	16.4±1.4	5.2±0.3 *
Oct4+ (%)	7.2±0.7	7.5±1.3	2.0±0.5 *
Nanog+ (%)	3.7±0.3	3.5±0.4	2.0±0.2 **
Sox2+ (%)	1.3±0.3	1.3±0.4	0.3±0.1 *
ABCB5+ (%)	31.3±1.6	28.1±1.1	11.6±2.9 *

Hep3B cells were treated with anti-GEP monoclonal antibody (A23), mouse IgG isotype antibody (mIgG) (antibodies at 50 µg/ml) or without antibody (control, CTL) for 24 h. Protein expression of GEP, β-catenin, Oct4, Nanog, Sox2 and ABCB5 was measured by intracellular staining and analyzed by flow cytometry. Data are expressed as mean percentage of positive cells ± SD from three independent experiments.

*P<0.05,

**P<0.01,

***P<0.001 when compared with Hep3B control.

### GEP-expressing cells possess CSC properties *in vitro*

**Figure 2:**
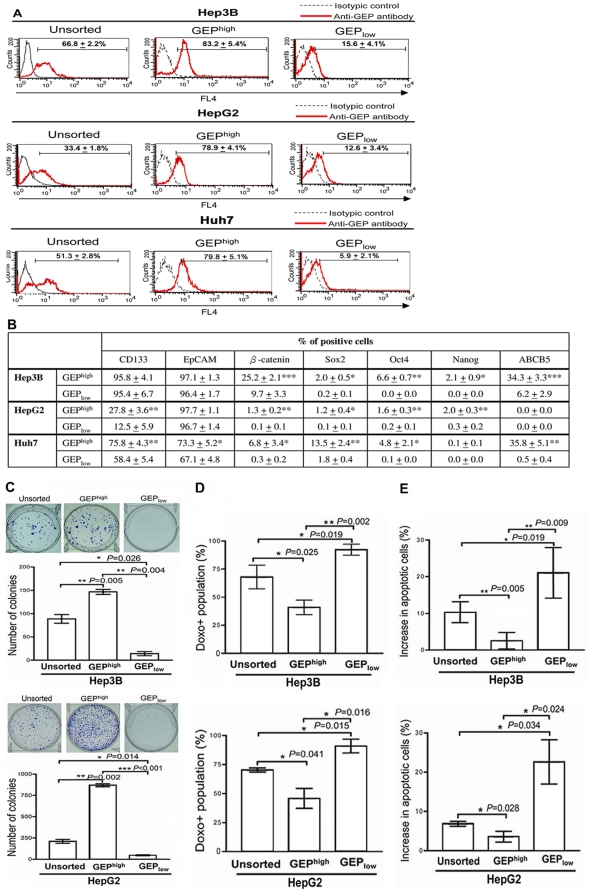
GEP-expressing cells isolated from liver cancer cell lines possess higher tumorigenic potential *in vitro*. (A) GEP expression in unsorted Hep3B, HepG2 and Huh7 cells, and the freshly isolated GEP^high^, and GEP_low_ subpopulations after cell sorting. Cells were sorted based on surface expression of GEP, but not on its intracellular expression, in order to keep the cells viable for subsequent functional assays. After cell isolation, 2 million of each sorted population was collected to assess the cell viability by trypan blue staining and the purity of the sorted subpopulations by flow cytometry using a different anti-GEP antibody (recognizing distinct epitopes compared to the antibodies used for cell sorting). Data are expressed as mean percentage of GEP+ cells ± SD. (B) Protein expression levels of stem cell markers β-catenin, Oct4, Nanog, Sox2 and ABCB5 in the sorted subpopulations were measured by intracellular staining and flow cytometric analysis. Data are expressed as mean percentage of positive cells ± SD. *P<0.05, **P<0.01, *** *P*<0.001 when compared with GEP_low_ cells. (C) Colony formation efficiencies of GEP^high^ subpopulations were higher than their respective GEP_low_ and unsorted Hep3B and HepG2 cells. (D, E) After exposure to doxorubicin (0.5 µg/ml for 24 h), GEP^high^ subpopulations retained significantly less doxorubicin and fewer cell apoptosis than GEP_low_ and unsorted Hep3B and HepG2 cells. *P<0.05, **P<0.01, *** *P*<0.001 when compared with unsorted controls.

To better characterize the stem cell properties of GEP-expressing cells, GEP^high^ and GEP_low_ subpopulations were isolated from human liver cancer cell lines Hep3B, HepG2 and Huh7, and examined for their expression of stem cell markers using flow cytometry. Cells were sorted based on the surface expression of GEP, but not on its intracellular expression, because permeabilization was not feasible to keep the cells viable for subsequent functional assays. After cell isolation, 2 million of each sorted population was collected to assess the cell viability by trypan blue staining and purity by flow cytometry using a different anti-GEP antibody that recognized distinct epitopes from those antibodies used for cell sorting. The purity of the GEP^high^ and GEP_low_ subpopulations were about 80% to 90%, respectively, as revealed by post-sorting analysis (Figure 2A). GEP^high^ cells were found to express higher levels of ES cells-related signaling molecules β-catenin, Oct4, Nanog, Sox2 and ABC drug transporter ABCB5 than GEP_low_ counterparts in the three HCC cell lines (Figure 2B). For hepatic CSC surface marker CD133, higher surface expression in GEP^high^ cells than GEP_low_ counterpart was found in HepG2 and Huh7, but not Hep3B, which was probably due to the extremely high level of CD133 (>95%) constitutively expressed in the Hep3B cells (data not shown). For EpCAM, higher surface expression was observed in GEP^high^ than GEP_low_ populations in Huh7, but not Hep3B and HepG2, in which majority of cells (>95%) were found to express EpCAM. The data is therefore consistent with the previous findings that GEP-expressing cells associated with stem cell marker expressions compared with the GEP negative cells.

To determine whether GEP-expressing cells were enriched for CSCs, we compared the tumorigenic potential and stem cell properties of GEP^high^ cells with their counterparts *in vitro* in Hep3B and HepG2 cells, which represent high and low GEP-expressing cell lines, respectively. Clonogenicity of the cells was assessed by colony formation assay. GEP^high^ Hep3B cells were able to form significantly more colonies compared with the GEP_low_ cells and the unsorted control. Similar observation was shown with an independent liver cancer cell HepG2 (Figure 2C).

To examine the role of GEP in chemoresistance, cells were incubated with doxorubicin and assessed for intracellular drug accumulation and cell apoptosis. GEP^high^ subpopulations, in both Hep3B and HepG2 cells, demonstrated significantly lower doxorubicin uptake (Figure 2D) and cell apoptosis (Figure 2E) when compared with unsorted control; while GEP_low_ subpopulations showed increased doxorubicin uptake and cell apoptosis when compared with unsorted populations.

### GEP^high^ABCB5+ cells demonstrate higher colony formation ability and chemoresistance

According to CSC hypothesis, CSCs represent only a rare population of the tumor [4]. This implies that GEP^high^ subpopulation is still heterogeneous, and possibly consists of subsets with differential tumorigenic potentials. Therefore, we sought to further dissect the GEP^high^ population by additional co-expressing marker.

**Figure 3:**
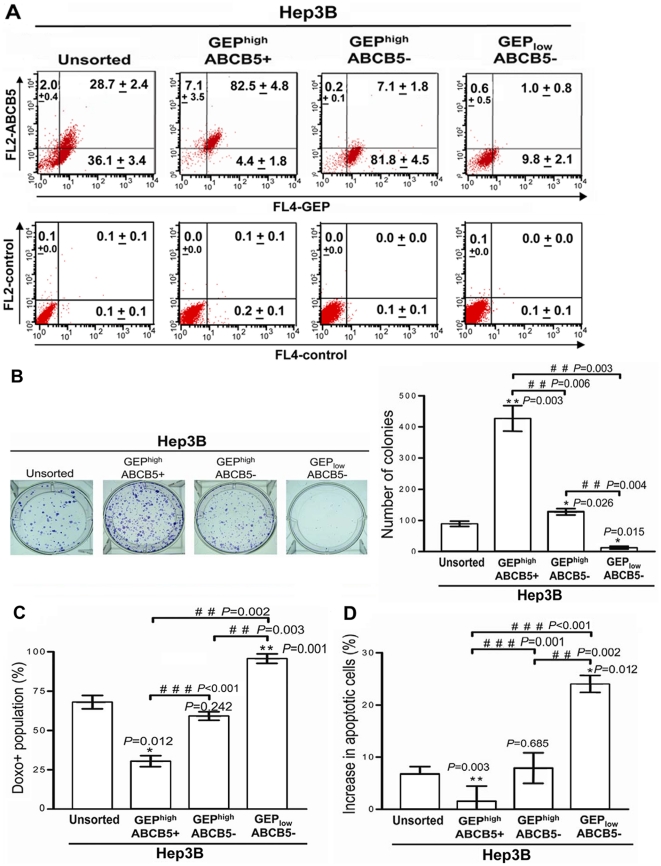
Co-expression of GEP and ABCB5 further enriches a hepatic CSC subpopulation. (A) GEP and ABCB5 expression in unsorted Hep3B cells, and the freshly isolated GEP^high^ABCB5+, GEP^high^ABCB5− and GEP_low_ABCB5− subpopulations. Cells were sorted based on surface expression of GEP and ABCB5. After cell isolation, 2 million of each sorted subpopulation was collected to assess purity by flow cytometry using different anti-GEP and anti-ABCB5 antibodies that recognized epitopes distinct from those of the antibodies used for cell sorting. Note that most ABCB5+ cells were also GEP+, thus there was no GEP_low_ABCB5+ cells (left panel). Mean percentage of cells ± SD is shown in each quadrant. (B) Colony formation efficiencies of GEP^high^ABCB5+ subpopulation were higher than GEP^high^ABCB5−, GEP_low_ABCB5− and unsorted cells. (C, D) After exposure to doxorubicin (0.5 µg/ml for 24 h), GEP^high^ABCB5+ cells retained significantly less doxorubicin and fewer cell apoptosis than the other subpopulations. *P<0.05, **P<0.01 when compared with unsorted controls; # *P*<0.05, # # *P*<0.01, # # # *P*<0.001 between groups denoted by horizontal lines.

In Hep3B cells, the majority of ABCB5+ cells expressed GEP, but only a subset of GEP+ cells were ABCB5+ (Figure 3A, left panel). In addition, we have shown recently that ABCB5 regulated hepatic cancer stem cell markers CD133 and EpCAM [21]. We therefore hypothesized that GEP in combination with ABCB5 might define more accurately the hepatic CSC population.

We isolated GEP^high^ABCB5+, GEP^high^ABCB5− and GEP_low_ABCB5− cells from Hep3B. Cells were sorted based on the surface expression of GEP and ABCB5, but not on their intracellular expression, because permeabilization was not feasible to keep the cells viable for subsequent functional assays. After cell isolation, sorted subpopulations were assessed for cell viability by trypan blue staining and purity by flow cytometry using different anti-GEP or anti-ABCB5 antibodies that recognized distinct epitopes from those antibodies used for cell sorting. At least 80% purity was obtained in each subpopulation (Figure 3A) and their tumorigenic potentials were examined both *in vitro* and *in vivo*.

GEP^high^ABCB5+ cells demonstrated induction of the largest number of colonies, while the GEP^high^ABCB5− cells showed intermediate ability to form colonies but still significantly higher than the unsorted control. Importantly, GEP_low_ABCB5− subpopulation was almost unable to form colonies (Figure 3B).

Upon exposure to doxorubicin, GEP^high^ABCB5+ cells demonstrated significantly lower level of doxorubicin uptake and cell apoptosis when compared with GEP^high^ABCB5− cells, and unsorted control. On the contrary, GEP_low_ABCB5− cells were highly sensitive to doxorubicin in terms of drug accumulation and cell apoptosis (Figure 3C and D)

### GEP^high^ABCB5+ cells are more tumorigenic *in vivo*

**Figure 4:**
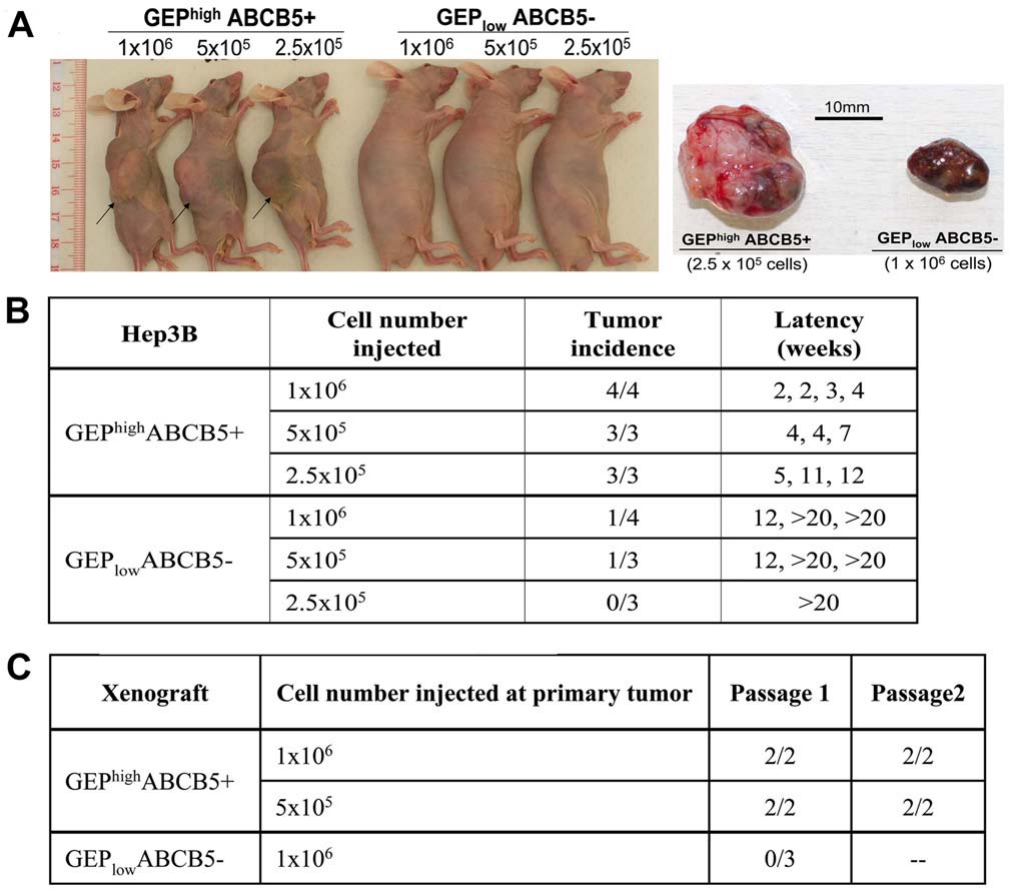
GEP^high^ABCB5+ Hep3B cells possess enhanced tumorigenic potential *in vivo*. (A) Nude mice injected subcutaneously with GEP^high^ABCB5+ and GEP_low_ABCB5− cells after 8 weeks. The right panel shows the subcutaneous tumors derived from 2.5×10^5^ GEP^high^ABCB5+ and 1×10^6^ GEP_low_ABCB5− cells at week 16. (B) Tumorigenicity of GEP^high^ABCB5+ and GEP_low_ABCB5− cells. (C) Serial transplantation of primary tumors generated from GEP^high^ABCB5+ and GEP_low_ABCB5− cells.

Tumor development experiments using GEP^high^ABCB5+ and GEP_low_ABCB5− subpopulations isolated from Hep3B cells were performed in immunocompromised nude mice. All mice injected with GEP^high^ABCB5+ cells developed tumors. Mice injected with 1×10^6^ double positive cells formed tumors within 2 to 4 weeks after inoculation. On the contrary, only 2/10 mice injected with GEP_low_ABCB5− cells generated relatively small tumors and required longer latency (12 weeks) (Figure 4A and B).

To demonstrate the *in vivo* self-renewal ability of the cells, xenografts growing from the initial inoculation were serially transplanted. Serial transplantation of xenografts derived from GEP^high^ABCB5+, but not GEP_low_ABCB5− subpopulation, generated tumor nodules in the second and subsequently the third passage in nude mice (Figure 4C).

We then characterized the xenograft tumors formed by GEP^high^ABCB5+ cells by flow cytometry, and these cells revealed low levels of GEP (8.3±5.2%) and ABCB5 (6.6±5.3%). The phenomenon that transplantation of GEP^high^ABCB5+ cells resulted in a heterogeneous populations consisting of GEP and ABCB5 positive and negative cells suggested that tumor hierarchy existed, in which the GEP+ and ABCB5+ cells were able to self-renew and differentiate, generating their positive and negative counterparts in the tumor mass (Figure S5). To validate the self-renewal ability of GEP and ABCB5 expressing cells, we performed *in vitro* short-term passages of cells derived from the GEP^high^ABCB5+ xenograft. Over the 3 week culture period, asymmetric division occurred in which GEP+ and ABCB5+ fractions increased until the levels returned to those of the unsorted Hep3B cells (Figure S5).

The above data was therefore consistent with the *in vitro* findings that co-expression of GEP and ABCB5 could enrich a subpopulation with higher tumorigenic potential than their counterparts.

### Phenotypic characterization of GEP-expressing cells in HCC clinical specimens

**Figure 5:**
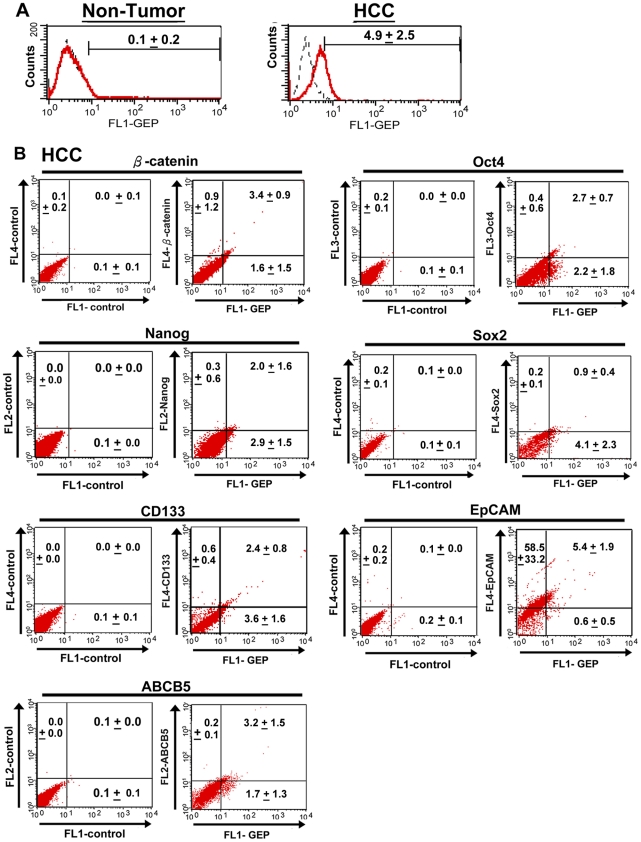
Phenotypic characterization of GEP-expressing cells in HCC clinical specimens. Liver tissues obtained from HCC patients were digested into single cell suspension, and assessed for the expression of albumin, GEP and stem cell markers. (A) Flow cytometric analyzes showing GEP expression in HCC, but not in adjacent non-tumor liver tissues. GEP expression analysis was performed by dual-color flow cytometry, gating on the albumin+ hepatocytes of liver specimens. (B) Co-expression of GEP and stem cell markers was performed by triple-color flow cytometry, gating on the albumin+ hepatocytes of HCC specimens. Protein expression of GEP, β-catenin, Oct4, Nanog, Sox2 and ABCB5 was measured by intracellular staining, while that of CD133 and EpCAM was assessed by surface staining. Cells co-expressing the respective markers were shown in the upper right quadrant of dot plots. Data were presented as mean percentage of cells ± SD.

To confirm the stem cell phenotype of GEP-expressing cells in clinical settings, GEP was co-stained with the aforementioned stem cell markers in six HCC clinical specimens. Liver tissues were digested into single cell suspension, and assessed for the expression of albumin, GEP and the stem cell markers by flow cytometry. Consistent with our previous findings, GEP protein expression was observed in HCC (4.9±2.5%), but not in adjacent non-tumor liver tissues (Figure 5A). Co-expression of GEP and stem cell markers was examined by triple-color flow cytometry, gating on the albumin+ hepatocytes. ES cells-related signalling molecules including β-catenin, Oct4, Nanog, Sox2 and hepatic CSC surface marker CD133 was preferentially expressed in GEP+ population (Figure 5B). EpCAM, it was found to express in the majority of hepatocytes (63.8±34.2%), and most, if not all GEP+ cells were also positive for EpCAM. Most importantly, the majority of the cells expressing ABCB5 were also positive for GEP staining, but only a subset of GEP+ cells were ABCB5+. The result is therefore consistent with our *in vitro* findings on the co-expression of GEP and ABCB5, and provided further evidence that co-expression of GEP and ABCB5 might define more accurately the hepatic CSC population.

## Discussion

Previously, our group reported the role of GEP in HCC growth and tumorigenicty [19], regulation of chemoresistance [25], and the potential to serve as therapeutic target [20]. However, its exact biological role in human liver remains unknown. In this study, we demonstrate that GEP is a hepatic oncofetal protein with the properties of both primitive stem and cancer cells. The present work is the first report showing the nature of GEP as oncofetal protein in human and mouse livers, and also the first study showing the CSC properties of GEP-expressing cells. Therefore, GEP might be a promising candidate to pursue further for novel therapeutic development for liver cancer.

Since tumorigenicity of CSCs is largely determined by their self-renewal ability [26], differentiation of CSCs might therefore suppress carcinogenesis. It is now believed that multiple dysregulated self-renewal pathways are functioning to maintain the CSC subpopulations [27]. Our transfection studies demonstrated a positive correlation between the expression of GEP and those of stem cell signaling molecules including β-catenin, Oct4, Nanog and Sox2, suggesting a regulatory role of GEP on these signaling molecules. Dysregulation of the Wnt/β-catenin was found to result in transformation of liver stem/progenitor cells, thereby implying a role in self-renewal of hepatic stem cells [28]. Oct4, Nanog and Sox2 are three core transcription factors regulating cellular pluripotency and are known to suppress differentiation in ES cells [29]. In this study, we showed that GEP blockage by anti-GEP monoclonal antibody A23 could significantly suppress the expression of these pluripotency-related signaling molecules. Our group previously showed that phosphorylation of Akt were reduced after A23 treatment in a dose-dependent manner [20]. PI3K/Akt plays crucial role in proliferation, survival, and maintenance of pluripotency in ES cells [30], [31], and these effects were found to mediate through the phosphorylation of GSK3β, leading to increased Wnt/β-catenin activity [32], [33]. Recently, Lee *et al* reported that Wnt and PI3K signals were required for the expression of representative pluripotency marker genes such as Oct4 and Sox2 [33]. Therefore, it is possible that GEP might regulate the expression of the aforementioned pluripotency-related signaling molecules via the activation of PI3K/Akt pathway, although further investigations are needed to support this hypothesis. While the underlying regulatory mechanism remains to be elucidated, the data suggest that GEP not only define a CSC subpopulation in HCC, but also functionally contribute to the stemness of CSCs by regulating the related signalling molecules. Targeting GEP by antibody A23 might therefore serve as a potential therapeutic tool to “de-stem” the CSCs and ultimately sensitize them to conventional therapies. Further efforts would be made towards elucidating the molecular mechanism(s) underlying the regulatory role GEP in maintaining pluripotency of CSCs.

Increasing evidence has shown that a single marker is insufficient to define the real CSC population [34]. It is therefore worthwhile to identify additional markers co-expressing with GEP to better characterize the hepatic CSCs. Recently, we have demonstrated a positive correlation between GEP and ABCB5 and also their roles in chemoresistance in HCC [25]. While ABCB5+ cells were reported to define a CSC subpopulation in melanoma [35], their tumorigenic potential in HCC remained unknown. Here, we provide further evidence showing that GEP and ABCB5 could define more specifically a hepatic CSC population with greater tumorigenic potential and stem cell properties than GEP alone. Since ABCB5 represents a potential chemoresistance mechanism [36], GEP^high^ABCB5+ hepatic CSCs might be responsible for both initiation and chemotherapeutic refractoriness of HCC. Together with the regulatory role of GEP in stem cell-related signalling molecules, targeting GEP and ABCB5 might therefore represent novel therapeutic strategies to the disease by interrupting both chemoresistance and pluripotency in the CSCs.

GEP^high^ABCB5+ cells constitute about 4% of hepatocytes in HCC clinical specimens (Figure 5). According to CSC hypothesis, tumor-initiating ability specifically resides in a rare population of cells within a tumor [4]. However, it is now believed that the frequency of CSCs could vary dramatically within cancer of the same type [35], [37]. In fact, recent mathematical analyses indicated that the CSC proportion should be higher in more aggressive tumors [38]. Nevertheless, continued phenotypic studies of GEP^high^ABCB5+ cells in HCC clinical specimens are needed to more accurately determine the CSC frequency and to prove enrichment, which might eventually allow correlation between CSC frequency, tumor grade and clinical outcome. The current study has a small sample size therefore with limited statistical power to analyze for the clinicopathological characteristics. Further validation in clinical settings is needed to confirm the tumor-initiating ability of GEP^high^ABCB5+ subpopulation.

## Supporting Information

Figure S1 **GEP expression in mouse hepatocytes by flow cytometry.** Flow cytometric analyzes demonstrating GEP, albumin and AFP expression in mouse livers. GEP expression in (A) total cells isolated from mouse livers, (B) AFP+ and/or albumin+ cells, (C) AFP+ cells, and (D) albumin+ cells was performed by intracellular staining and single- or dual-color flow cytometry. (E) AFP and (F) albumin expression in mouse livers at different developmental stages were examined by flow cytometry.
(TIF)

Figure S2 **Protein expression of GEP in mouse embryonic livers by Western blot using anti-GEP monoclonal antibody A23.** The anti-GEP monoclonal antibody A23 specifically recognized mouse GEP at about 75 kDa. GEP expression was detected in mouse embryonic livers and increased from E11.5 to E17.5. β-actin expression was examined to ensure equal loading of protein.
(TIF)

Figure S3 **Phenotypic characterization of GEP-expressing cells in liver cancer cell line Hep3B.** Flow cytometric analyzes showing co-expression of GEP with stem cell markers β-catenin, Oct4, Nanog, Sox2, CD133, EpCAM and ABCB5. Protein expression of GEP, β-catenin, Oct4, Nanog, Sox2 and ABCB5 was measured by intracellular staining, while that of CD133 and EpCAM was assessed by surface staining. Cells co-expressing the respective markers were shown in the upper right quadrant of each scatter plot. Data are expressed as mean percentage of cells ± SD.
(TIF)

Figure S4 **Phenotypic characterization of GEP-expressing cells in liver cancer cell line Huh7.** Flow cytometric analyzes showing co-expression of GEP with stem cell markers β-catenin, Oct4, Nanog, Sox2, CD133, EpCAM and ABCB5. Protein expression of GEP, β-catenin, Oct4, Nanog, Sox2 and ABCB5 was measured by intracellular staining, while that of CD133 and EpCAM was assessed by surface staining. Cells co-expressing the respective markers were shown in the upper right quadrant of each scatter plot. Data are expressed as mean percentage of cells ± SD.
(TIF)

Figure S5 ***In vitro*** **self-renewal ability of GEP positive and ABCB5 positive cells.** Flow cytometric analyzes showing the increasing GEP and ABCB5 expression in primary culture established from GEP^high^ABCB5+ cells-induced xenograft tumors over a 3-week culture period.
(TIF)
