# Supplementary figures and images for: Granulin-Epithelin Precursor Is an Oncofetal Protein Defining Hepatic Cancer Stem Cells
Source: PLoS One. 2011 Dec 16;6(12):e28246. doi: 10.1371/journal.pone.0028246 (PMC3241621; doi:10.1371/journal.pone.0028246)

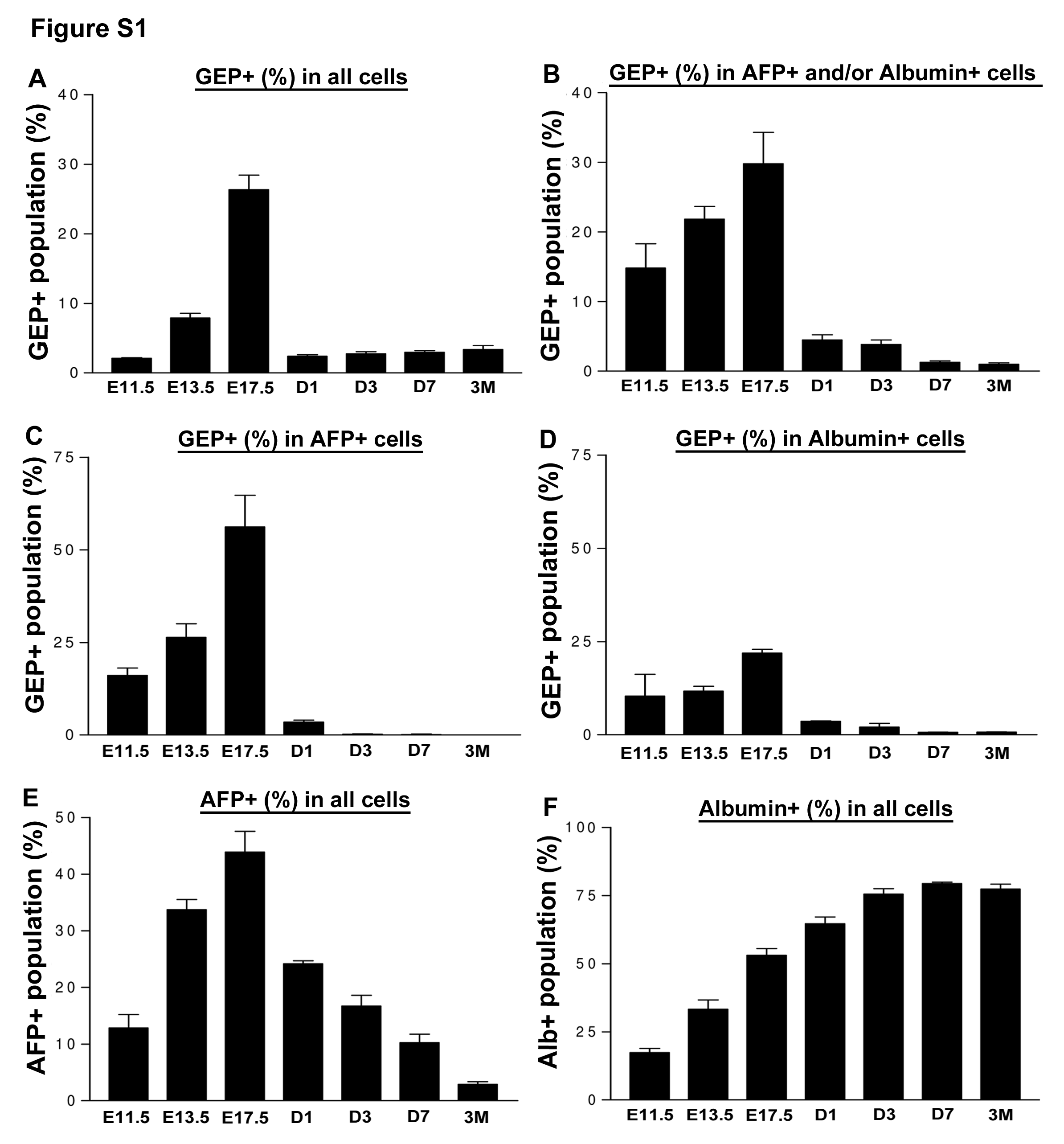

Supplement: Figure S1 — GEP expression in mouse hepatocytes by flow cytometry. Flow cytometric analyzes demonstrating GEP, albumin and AFP expression in mouse livers. GEP expression in (A) total cells isolated from mouse livers, (B) AFP+ and/or albumin+ cells, (C) AFP+ cells, and (D) albumin+ cells was performed by intracellular staining and single- or dual-color flow cytometry. (E) AFP and (F) albumin expression in mouse livers at different developmental stages were examined by flow cytometry. (TIF) [file pone.0028246.s001.tif]

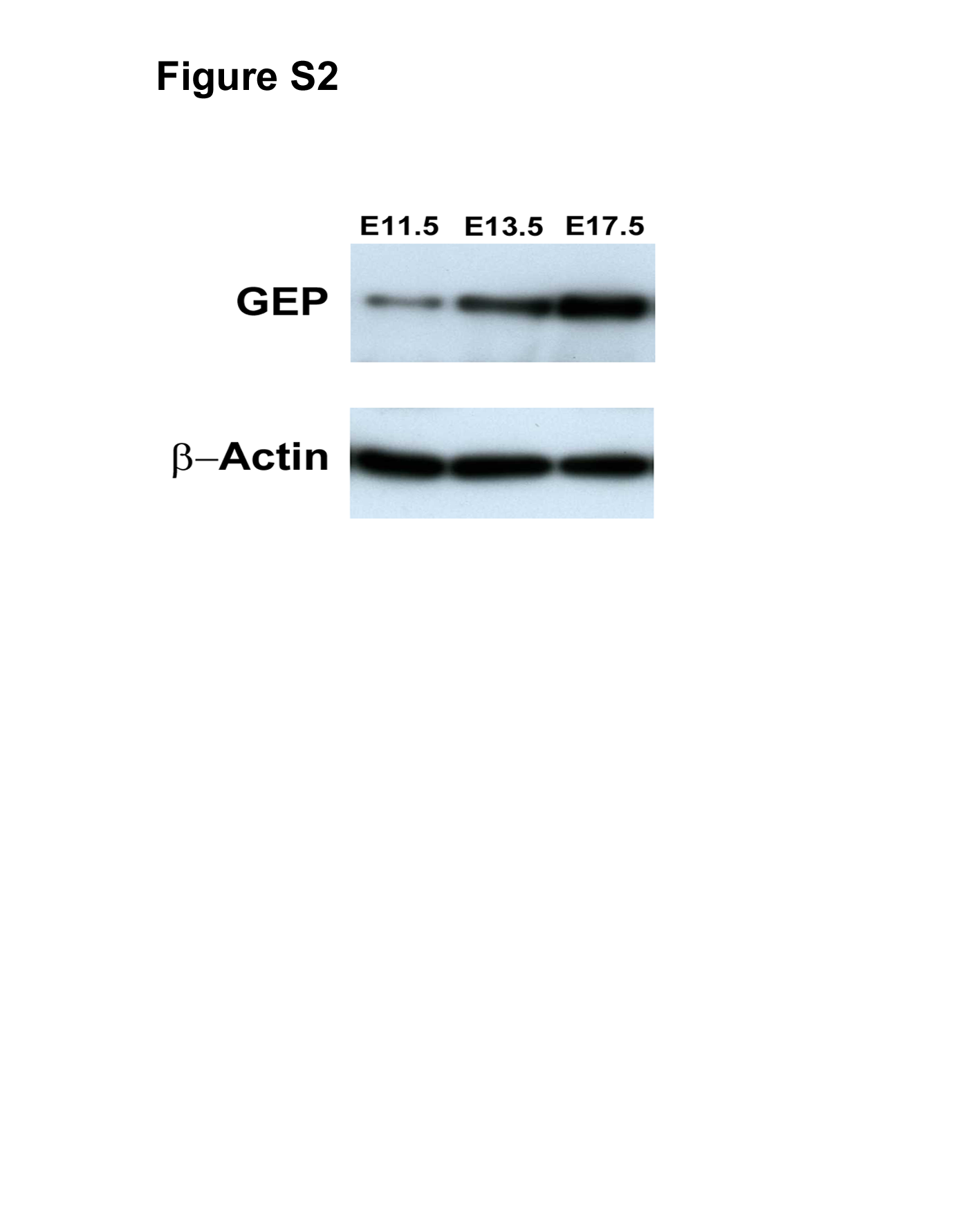

Supplement: Figure S2 — Protein expression of GEP in mouse embryonic livers by Western blot using anti-GEP monoclonal antibody A23. The anti-GEP monoclonal antibody A23 specifically recognized mouse GEP at about 75 kDa. GEP expression was detected in mouse embryonic livers and increased from E11.5 to E17.5. β-actin expression was examined to ensure equal loading of protein. (TIF) [file pone.0028246.s002.tif]

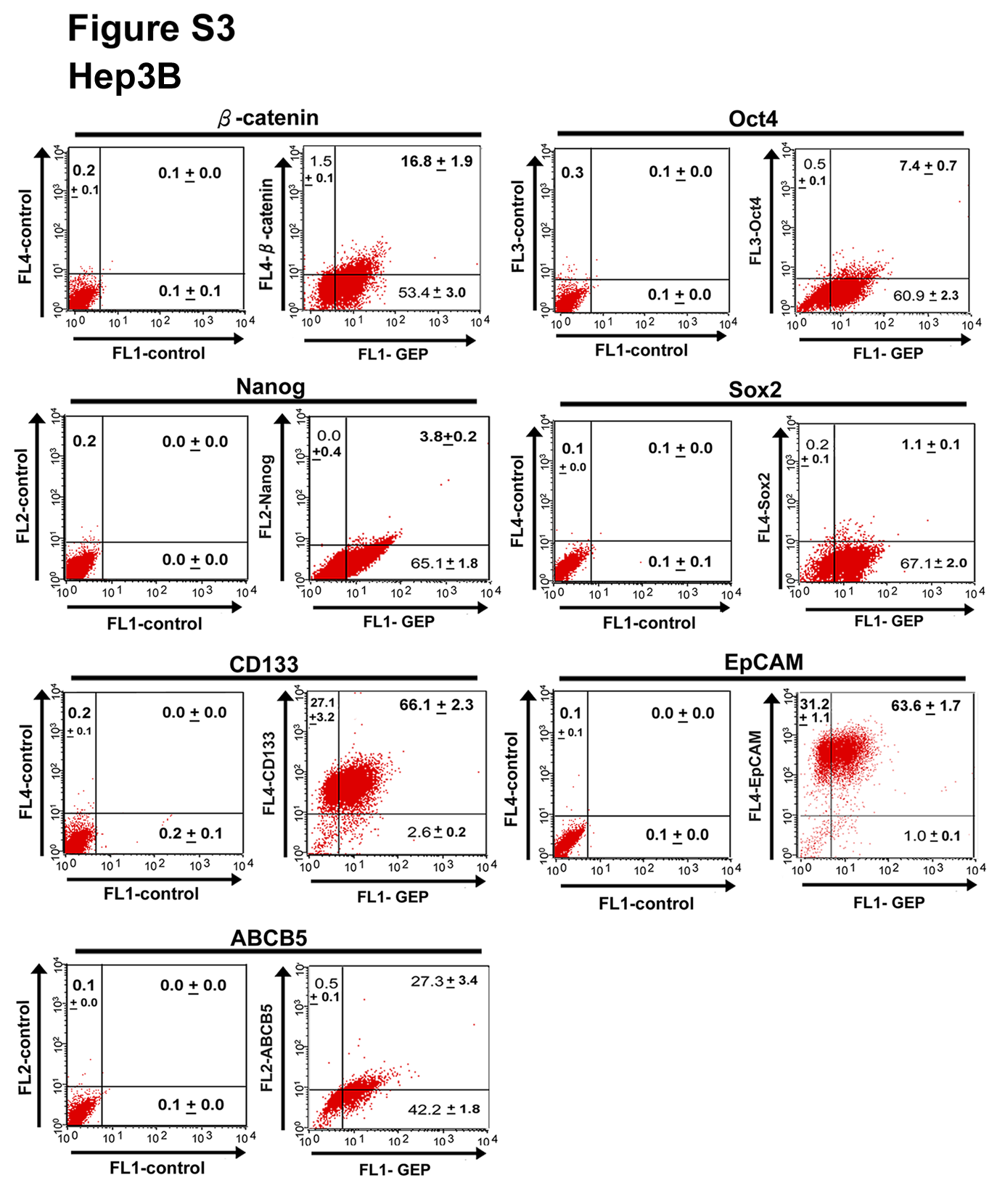

Supplement: Figure S3 — Phenotypic characterization of GEP-expressing cells in liver cancer cell line Hep3B. Flow cytometric analyzes showing co-expression of GEP with stem cell markers β-catenin, Oct4, Nanog, Sox2, CD133, EpCAM and ABCB5. Protein expression of GEP, β-catenin, Oct4, Nanog, Sox2 and ABCB5 was measured by intracellular staining, while that of CD133 and EpCAM was assessed by surface staining. Cells co-expressing the respective markers were shown in the upper right quadrant of each scatter plot. Data are expressed as mean percentage of cells ± SD. (TIF) [file pone.0028246.s003.tif]

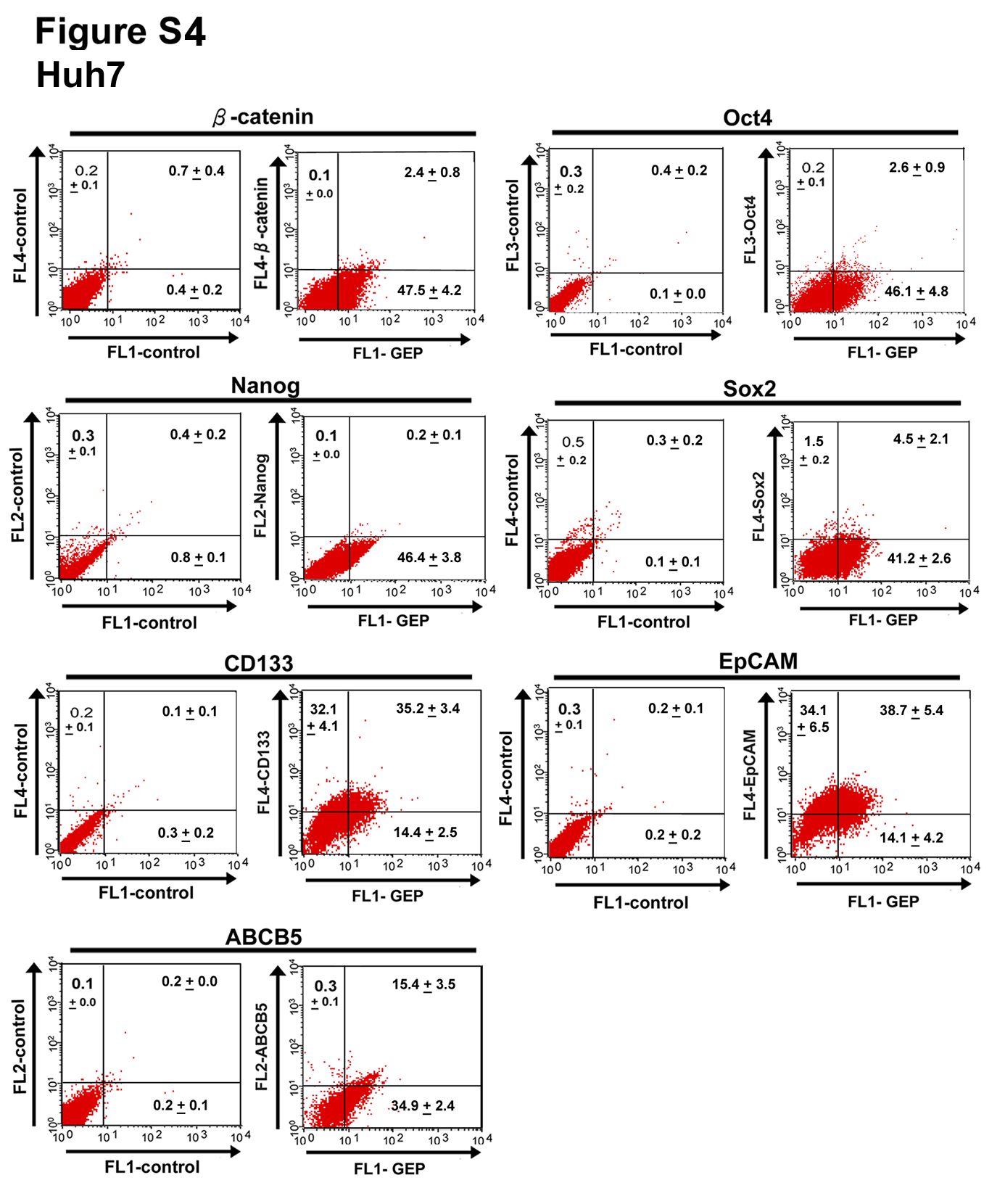

Supplement: Figure S4 — Phenotypic characterization of GEP-expressing cells in liver cancer cell line Huh7. Flow cytometric analyzes showing co-expression of GEP with stem cell markers β-catenin, Oct4, Nanog, Sox2, CD133, EpCAM and ABCB5. Protein expression of GEP, β-catenin, Oct4, Nanog, Sox2 and ABCB5 was measured by intracellular staining, while that of CD133 and EpCAM was assessed by surface staining. Cells co-expressing the respective markers were shown in the upper right quadrant of each scatter plot. Data are expressed as mean percentage of cells ± SD. (TIF) [file pone.0028246.s004.tif]

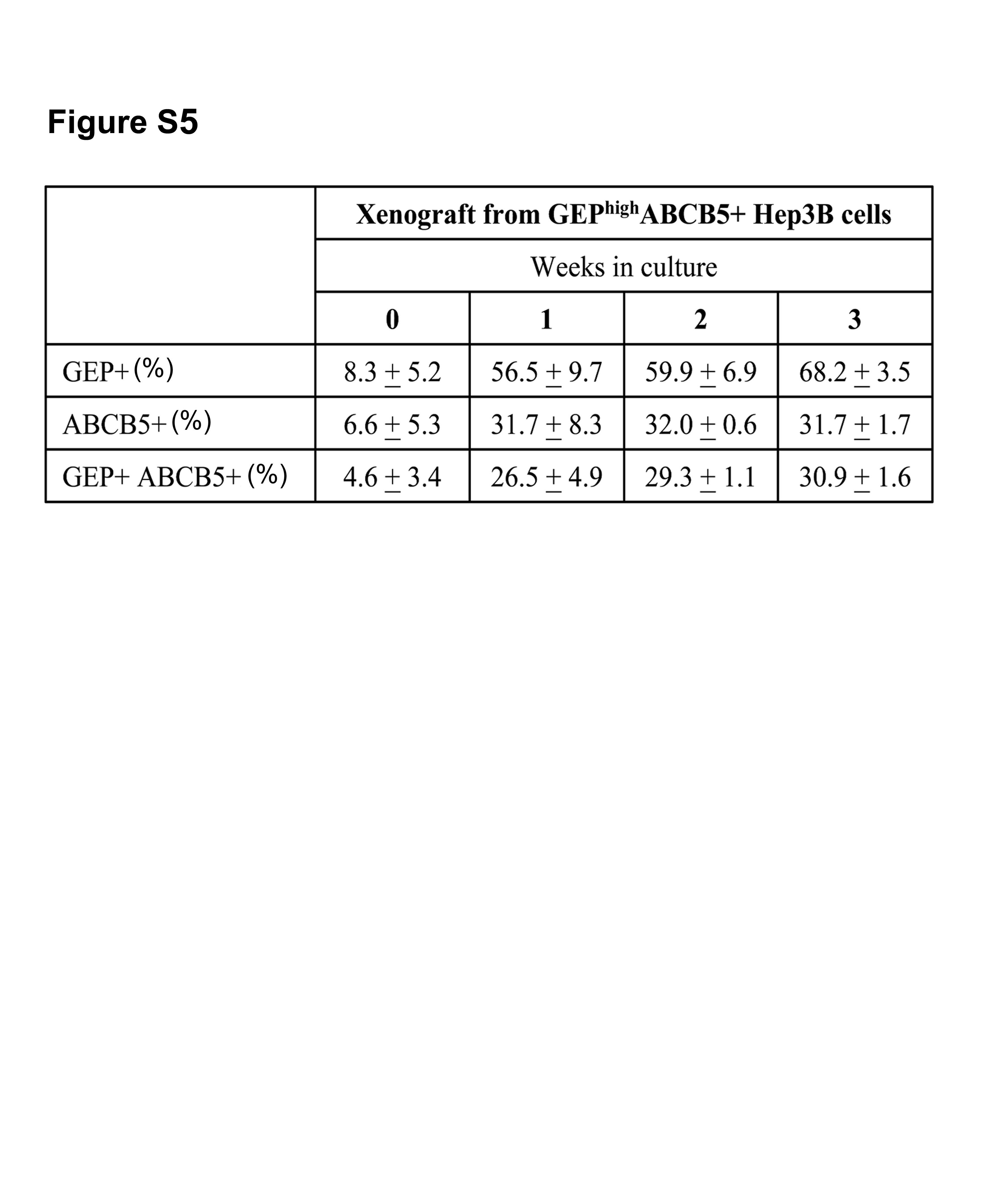

Supplement: Figure S5 — In vitro self-renewal ability of GEP positive and ABCB5 positive cells. Flow cytometric analyzes showing the increasing GEP and ABCB5 expression in primary culture established from GEPhighABCB5+ cells-induced xenograft tumors over a 3-week culture period. (TIF) [file pone.0028246.s005.tif]
